# Primary Yolk Sac Tumor of the Endometrium: A Case Report and Comprehensive Literature Review

**DOI:** 10.1155/crip/5517722

**Published:** 2026-06-19

**Authors:** Hao Cheng, Yan Song, Su-sheng Shi

**Affiliations:** ^1^ Department of Pathology, National Cancer Center/National Clinical Research Center for Cancer/Cancer Hospital, Chinese Academy of Medical Sciences and Peking Union Medical College, Beijing, China, cacms.ac.cn; ^2^ Department of Pathology, National Cancer Center/National Clinical Research Center for Cancer/Cancer Hospital & Shenzhen Hospital, Chinese Academy of Medical Sciences and Peking Union Medical College, Shenzhen, China, cacms.ac.cn

**Keywords:** diagnosis, endometrium, prognosis, treatment, yolk sac tumor (YST)

## Abstract

Yolk sac tumor (YST) of the endometrium is very rare, with fewer than 40 cases reported in the English literature. We here describe a case of primary endometrial YST and discuss the clinicopathological features with a literature review. A 66‐year‐old Chinese woman presented with abnormal vaginal bleeding for 15 days and a uterine mass for 3 days. The preoperative alpha‐fetoprotein (AFP) level was 9652.0 ng/mL, while other serum tumor markers, including carcinoembryonic antigen (CEA), neuron‐specific enolase (NSE), and *β*‐human chorionic gonadotropin (*β*‐HCG), were 40.2 ng/mL, 29.19 ng/mL, and 27.7 mIU/mL, respectively. Pelvic ultrasound imaging revealed a 7.1 × 6.6 × 6.0 cm mass in the endometrial cavity. The patient underwent total abdominal hysterectomy, salpingo‐oophorectomy, and partial omental resection. The morphologic and immunohistochemical pattern (cytokeratin+++, Sal‐Like Protein 4+++, AFP++, Focal Hepatocyte Nuclear Factor 1 beta+, and Focal Glypican‐3+) was consistent with a primary YST of the endometrium, and the final pathologic stage was IVb based on the International Federation of Gynecology and Obstetrics (FIGO) staging. Postoperative serum AFP level was 5193.0 ng/mL 5 days after the operation. Primary endometrial YST is extremely rare. It should be differentially diagnosed from other uterine malignancies. Complete surgical staging combined with chemotherapy may have a better survival impact on endometrial YST.

## 1. Introduction

Yolk sac tumor (YST), also known as an endodermal sinus tumor, is a malignant germ cell tumor (GCT) often accompanied by elevated alpha‐fetoprotein (AFP). YSTs account for about 10%–20% of all malignant ovarian GCTs [1]. The majority of YSTs arise in the gonads and the midline of the body of children and young women [2]. Approximately 10% YSTs occurred in extragonadal sites for female patients, such as the sacrococcygeal region, retroperitoneum, mediastinum, pineal gland, stomach, liver, omentum, pelvis, and genital system [3]. Primary YST arising in the endometrium is extremely rare. Here, we report a case of primary endometrial YST and discuss the clinicopathological features in conjunction with a literature review.

## 2. Case Report

In early June 2021, a 66‐year‐old Chinese woman (gravida 1, para 0) was admitted with abnormal vaginal bleeding for 15 days. The patient had menarche at 15 years old with 7 days of menstrual period and 30 days of cycle. The patient had a history of an aborted pregnancy and was naturally menopausal at 52 years old, with no abnormal vaginal discharge after menopause. On June 7, 2021, she underwent a routine physical examination at a local hospital due to persistent bleeding. Serum tumor marker testing, which was ordered by the attending gynecologist as part of a comprehensive workup for an atypical pelvic mass, revealed AFP 1210.0 ng/mL (reference value: 0–7.0 ng/mL) and carcinoembryonic antigen (CEA) 19.3 ng/mL (reference value: 0–5.0 ng/mL). Re‐examination at the local hospital on June 18 showed an AFP level of 9652.0 ng/mL, a CEA level of 40.2 ng/mL, and a neuron‐specific enolase (NSE) level of 29.19 ng/mL (reference value: 0–12.5 ng/mL), and levels of cancer antigen 125 (CA125), CA19‐9, CA15‐3, and CA72‐4 were all within the normal range. A pelvic ultrasound performed on June 18 revealed the uterine mass, which measured approximately 7.9 × 7.6 × 6.8 cm with irregular margins and heterogeneous echoes. On June 21, 2021, the patient was referred to another local hospital for further evaluation. Gynecological examination revealed a married vulva and a smooth vagina with blood stains in the vagina. The uterus presents an anterior position, is enlarged to a size consistent with 15 weeks of pregnancy, and is of medium quality, with no tenderness. The degree of activity was fair, and no obvious abnormalities were noted in the double attachment area. Repeat laboratory examination at this hospital showed AFP 8762.8 ng/mL, CEA 71.4 ng/mL, and *β*‐HCG 27.7 mIU/mL (reference value: 0–3.1 mIU/mL). Positron emission tomography/computed tomography (PET/CT) performed on June 25 showed multiple metastases in both lungs and multiple bone metastases in the sacrum, left ilium, left pubis, and right ischia.

**Figure 1:**
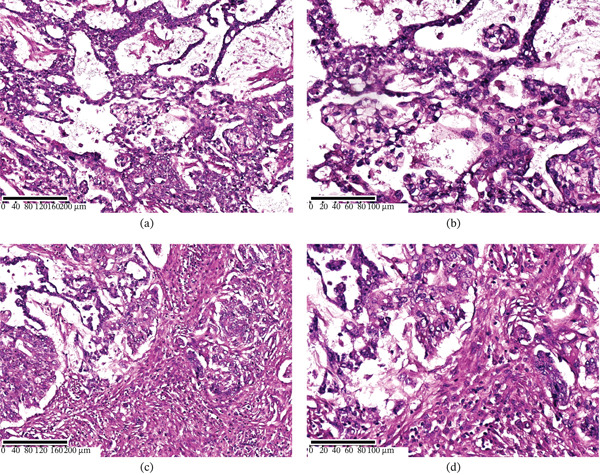
Pathological studies confirmed the diagnosis of endometrial yolk sac tumor (YST). (a) Magnification, ×100. (b) Magnification, ×200. (c) Magnification, ×100. (d) Magnification, ×200.

**Figure 2:**
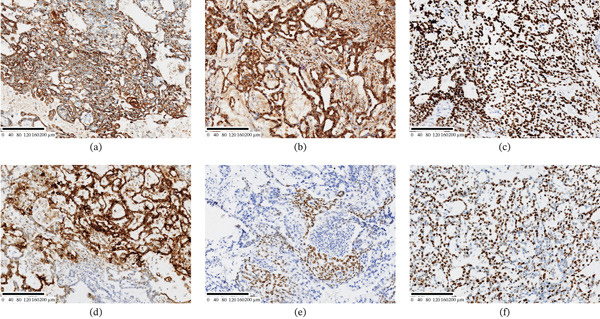
Immunohistochemistry assays (magnification, ×100) suggested (a) positive cytokeratin (CK), (b) positive alpha‐fetoprotein (AFP), (c) Positive Sal‐Like Protein 4 (SALL4), (d) Focal Positive Glypican‐3 (GPC‐3), (e) Focal Positive Hepatocyte Nuclear Factor 1 beta (HNF1*β*), and (f) Ki‐67 was positive in 60% cells.

Hysteroscopy was performed on June 22, 2021. Postoperative pathology showed (intrauterine tissue) a poorly differentiated malignant tumor, and it is considered a clear cell carcinoma, but YST is not excluded. Immunohistochemistry (IHC) showed cluster of differentiation 30 (CD30)−, focal AFP+, focal P40+, Sal‐Like Protein 4 (SALL4)+, NapsinA−, Focal Hepatocyte Nuclear Factor 1 beta (HNF1*β*)+, Focal Glypican‐3 (GPC‐3)+, estrogen receptor (ER)−, progesterone receptor (PR)−, P53+++, cytokeratin (CK)+, focal vimentin+, and P504S−, and Ki‐67 has a positivity rate of approximately 90%. A total laparoscopic hysterectomy, bilateral salpingo‐oophorectomy, and partial omentectomy were performed on July 2, 2021. The patient was subsequently referred to our institution (National Cancer Center, Beijing) for a pathology consultation on July 13, 2021. Pathological results showed an endometrial malignant tumor, and it is considered to be YST combined with morphology and IHC (Figure 1). Microscopically, the tumor had the typical features of a YST with reticular and solid patterns. The reticulum comprised a labyrinth of channels lined by primitive cells expanding to form microcysts with flattened, clear, atypical epithelial cells. Hyaline globules were observed in the cells, and any other type of GCT or somatic carcinoma components was not found in the pure endometrial YST. A large number of blood vessel invasions were seen, and no nerve invasion was seen. The tumor involved more than 1/2 of the muscle wall but did not involve the serosal membrane. The tumor did not involve the cervix and parauterine, while the bilateral fallopian tubes and ovarian tissues were not significantly abnormal. There was no tumor involvement in omental adipose tissue. IHC showed vimentin−, CK+++, P40−, ER−, PR−, focal HNF1*β*+, NapsinA−, P53 was positive in 40% cells, P504S−, SALL4+++, focal GPC‐3+, AFP++, and CD30−, and Ki‐67 was positive in 60% cells (Figure 2).

### 2.1. Follow‐Up

Following the surgery on July 2, 2021, the patient was discharged and advised to undergo adjuvant chemotherapy. However, she did not return to our hospital for further treatment. Despite multiple attempts by our department to contact the patient and her family by telephone after her pathology consultation on July 13, 2021, we were unable to obtain further follow‐up information. Consequently, the patient was lost to follow‐up, and her subsequent outcome and survival status remain unknown.

## 3. Discussion

YSTs are highly malignant GCTs that mostly occur in the gonads, such as the ovary or testis [4]. A primary YST of the endometrium is extremely rare [5]. There is no widely accepted conclusion about the histogenesis of primary endometrial YST. There are currently four proposed mechanisms to explain how a germ cell neoplasm can arise in the endometrium [1]: (1) the abnormal migration of primordial germ cells, which are hidden in the endometrial basal layer, during embryogenesis, (2) a discovered ovarian YST metastasis, (3) tumor development from the embryonic tissue remaining in the uterus after an incomplete abortion, and (4) the abnormal differentiation of somatic cells. One of the more recognized theories is that primordial germ cells are left behind during reproduction and migration from the yolk sac endoderm in embryonic development, and primordial germ cells then gradually become tumors, leading to the occurrence of extragonadal YST eventually. In our case, the third potential hypothesis can possibly be applied given her history of induced abortion.

Our case adds to the few published reports of primary endometrial YST. We searched the related literature using the following words: “yolk sac tumor,” “YST,” “endodermal sinus tumor,” “primary,” “endometrium,” and “endometrial” in PubMed by July 2025. Furthermore, references of selected literature were also retrieved to obtain all potentially relevant articles. After review of related English literature, only 35 cases of primary endometrial YST have been reported. All cases are summarized in Table 1. Among 35 cases of primary endometrial YST, the median age was 59 years (range 24–87 years). Our patient was 66 years old, which was similar to the results from previous cases. However, there were 11 cases [6] of endometrial YST from M.D. Anderson Cancer Center, a hospital that primarily serves an adult patient group, which possibly contributes to an older median age. Of the 35 cases, 19 were pure YST, and 16 coexisted with other tumor types such as endometrioid adenocarcinoma, serous carcinoma, and carcinosarcoma. It has been reported [7] that pure endometrial YST may arise from pluripotent germ cells, while endometrial YST with somatic tumors may originate from malignant pluripotent somatic stem cells or possibly via “neometaplasia” or “retrodifferentiation,” by which a differentiated cell transforms into a more primitive form. Consistent with the published literature, abnormal vaginal bleeding was the most common symptom, which aligns with the clinical feature of the present case. Moreover, AFP is an essential tumor marker elevated in the majority of patients with tumors containing a YST component. Among the 35 cases published previously, most patients presented with elevated AFP levels before or after surgery, whereas only two cases [2, 8] had normal serum AFP levels. Our case also had an elevated serum AFP, which was a significant biomarker for diagnosing primary YST and differentiating it from other diseases, including endometrial cancer and an undetermined pelvic mass. Most of the reported cases experienced a reduction in serum AFP after surgery, as did our patient (from 9652.0 ng/mL preoperatively to 5193.0 ng/mL 5 days postoperatively).

**Table 1:** Summary of clinicopathologic features of primary endometrial YST.

Cases	Year	Age (y)	Alpha fetoprotein (AFP) level, preoperation	Alpha fetoprotein (AFP) level, postoperation	Other abnormal serum tumor markers	Symptoms	Tumor size (cm)	Surgery	Histopathology	Chemotherapy	Radiotherapy	Stage	Follow‐up, mo	Outcome
1	2025	≥ 70	NR	NR	None	Abnormal vaginal Bleeding and recurrent nausea/emesis	NR	TAH, BSO, and LD	YST	VP16 and DDP, four cycles	Yes	IA	24	Brain metastasis
2	2024	42	> 1210 ng/mL	380.5 ng/mL (20 days after operation)	None	Abnormal vaginal Bleeding	5.9	TAH, BSO, and LD	YST	BLM, VP16, and DDP, six cycles	None	IIIA	13	AWD
3	2021	43	1465 *μ*g/mL	NR	None	Abnormal vaginal	4	TAH, BSO, LD, OMT, and APD	YST	BLM, VP16, and DDP, six cycles	None	IA	15	NED
						Bleeding, epigastric pain								
4	2021	35	9,152 ng/mL	231.7 ng/mL (2 weeks after operation)	CA72‐4 14.39 U/mL	Abnormal uterine bleeding and extended menstrual	NR	TAH, BSO, LD, and OMT	YST and mature teratoma (right adnexa)	BLM, VP16, and DDP, four cycles	None	IA	21	NED
					NSE 16.48 ng/mL	Period								
5	2021	73	NR	10.5 ng/mL (1 month after operation)	None	Abdominal distension and bloating	4	TAH, BSO, and LD	YST	VP16 and DDP, four cycles	None	IIIA	15	NED
6	2021	73	NR	NR	NR	Postmenopausal bleeding and pelvic cramping	3.5	TAH, BSO, and LD	YST and carcinosarcoma	None	None	NR	6	NED
7	2020	65	359 ng/mL (preoperation)	NR	None	Abnormal vaginal bleeding	7	TAH and BSO	YST, embryonal carcinoma, and immature teratoma	BLM, VP16, and DDP, three cycles	None	IA	15	NED
8	2019	68	NR	133.4 ng/mL (6 days after operation)	None	Abnormal Vaginal bleeding	3.3	TAH, BSO, LD, and OMT	YST	BLM, VP16, and DDP, six cycles	None	II	6	NED
9	2019	27	NR	1584 ng/mL (1 day after operation)	None	Abnormal vaginal bleeding	7	TAH, BSG, and LD	YST	DOC and CBP, six cycles	None	IA	14	NED
				1265 ng/mL (3 days after operation)										
10	2019	38	Normal	NR	CA125 58.5 U/mL	Prolonged menstruation and	2.5	TAH, BSO, LD, OMT, and APD	YST	BLM, VP16, and DDP, six cycles	None	IVB	24	NED
						Increased menstrual bleeding								
11	2017	71	NR	NR	NR	Abnormal vaginal bleeding	NR	Yes	YST, SC, and EC	NR	NR	IIIA	19	DOD
12	2017	55	NR	NR	NR	Abnormal vaginal bleeding	NR	Yes	YST and complex hyperplasia	Yes	Yes	II	16	DOD
13	2017	59	NR	NR	NR	Abnormal vaginal bleeding and uterine mass	NR	NR	YST and EC	Yes	None	IB	NR	LFT
14	2017	68	NR	NR	NR	Abnormal vaginal bleeding and uterine mass	NR	Yes	YST	Yes	None	IV	14	DOD
15	2017	77	NR	NR	NR	Abnormal vaginal bleeding and uterine mass	NR	NR	YST, EC, and UDC	NR	NR	IIIC	NR	LFT
16	2017	64	NR	NR	NR	Abnormal vaginal bleeding	NR	Yes	YST and EC	Yes	Yes	IIIA	23	DOD
17	2017	87	NR	NR	NR	Abnormal vaginal bleeding	NR	Yes	YST and EC	Yes	None	II	7	AWD
18	2017	61	NR	NR	NR	Abnormal vaginal bleeding	NR	Yes	YST	Yes	None	IA	8	AWD
19	2017	63	NR	NR	NR	Abnormal vaginal bleeding	NR	Yes	YST and malignant mixed Müllerian tumor	Yes	Yes	IIIC1	5	NED
20	2017	62	NR	NR	NR	Abnormal vaginal bleeding	NR	Yes	YST and SC	Yes	None	IB	30	AWD
21	2017	77	NR	NR	NR	Abnormal vaginal bleeding	NR	Yes	YST, SC, CCC, and UDC	Yes	None	IIIC2	17	AWD
22	2016	63	NR	244.6 IU/mL (6 weeks after operation)	NR	Postmenopausal	12	TAH, LSO, OMT, and APD	YST	BLM, VP16, and DDP, three cycles	None	IVB	6	AWD
				101.4 IU/mL (10 weeks after operation)		Bleeding								
23	2015	57	31,844 IU/mL	NR	NR	Abdominal pain	10.5	TAH, BSO, OMT, and LD	YST	BLM, VP16, and DDP	None	IVB	1	DOD
24	2015	44	> 30,000 IU/mL	NR	NR	Abnormal	19	TAH, BSO, OMT, and LD	YST	BLM, VP16, and DDP	None	IB	6	NED
						Vaginal bleeding								
25	2014	31	242.3 IU/mL	11.7 IU/mL (2 weeks after operation)	NR	Menorrhagia	4	TAH, BSO, LD, and OMT	YST	BLM, VP16, and DDP, three cycles	None	IA	24	NED
26	2013	28	1522 ng/mL	166.4 ng/mL (2 days after operation)	*β*‐hCG 518.9 mIU/mL	Abnormal vaginal bleeding	6	TAH, BSO, LD, OMT, and APD	YST and EC	PTX, ADM, DDP, CBP, VP16, MTX, BLM, Act‐D, VCR, FU, OXA, CPA, and PYM	None	IVB	10	AWD
					CA125 129 U/mL									
27	2011	29	3593.4 ng/mL	NR	None	Abnormal vaginal bleeding	6.7	MRH, LSO, and LD	YST	BLM, VP16, and DDP, four cycles	None	II	39	NED
28	2011	30	1.762 ng/mL	759.5 ng/mL (5 days after operation)	CA125 36	Abnormal	5.5	TAH	YST	BLM, VP16, and DDP, three cycles	None	II	72	NED
					U/mL	Vaginal bleeding								
29	2006	65	2306 ng/mL	NR	CA19‐9 50 U/mL and STN 110 U/mL	Watery discharge	7	MRH, BSO, and LD	YST and carcinosarcoma	PTX and CBP, five cycles	None	IIIc	NR	NED
30	2001	59	NR	27,670 U/mL (postoperation)	NR	Postmenopausal bleeding	NR	TAH, BSO, OMT, and LD	YST and EC	BLM, VP16, and DDP, six cycles	21 Gy by vaginal	IA	19	AWD
											Brachytherapy			
31	1998	49	NR	Normal (3 weeks after operation)	None	Abnormal vaginal bleeding	1.3	TAH, BSO, and LD	YST	None	45 Gy on the pelvis	IA	28	NED
32	1990	42	18,530 *μ*g/L	7100 *μ*g/L (4 days after operation)	None	Abnormal vaginal bleeding	6	TAH and BSO	YST	BLM, VLB, and DDP	None	IA	24	NED
33	1988	27	1580 ng/mL	NR	None	Menorrhagia	2.4	TAH, BSO, and OMT	YST	VCR, Act‐D, and CPA	None	IA	14	NED
34	1988	24	NR	3600 ng/mL (postoperation)	NR	Abdominal pain	10	SH and BSO	YST	VCR, Act‐D, and CPA	Yes	IVA	24	DOD
35	1980	28	NR	380 ng/mL (postoperation)	NR	Menorrhagia and pelvic pain	NR	TAH and BSO	YST	VCR, CTX, ADM, MTX, and FU	None	NR	8	DOD
Present	2025	66	9652.0 ng/mL	5193.0 ng/mL (5 days after operation)	CEA 40.2 ng/mL, NSE 29.19 ng/mL, and HCG 27.7 mIU/mL	Abnormal vaginal bleeding	7.1	TAH, BSO, and OMT	YST	None	None	IVB	1	NED
						Uterine mass								

Abbreviations: Act‐D, actinomycin D; ADM, adriamycin; APD, appendectomy; AWD, alive with disease; BLM, bleomycin; BSG, bilateral salpingectomy; BSO, bilateral salpingo‐oophorectomy; CBP, carboplatin; CCC, clear cell carcinoma; CPA, cyclophosphamide; DDP, cisplatin; DOC, docetaxel; DOD, dead from the disease; EC, endometrioid adenocarcinoma; FU, floxuridine; LD, lymphadenectomy; LFT, lost to follow‐up; LN, lymph node; LSO, left salpingo‐oophorectomy; MRH, modified radical hysterectomy; MTX, methotrexate; NED, no evidence of the disease; NR, not reported; OMT, omentectomy; OXA, oxaliplatin; PTX, paclitaxel; PYM, pingyangmycin; SC, serous carcinoma; SH, supracervical hysterectomy; TAH, total abdominal hysterectomy; UDC, undifferentiated carcinoma; VCR, vincristine; VLB, vinblastine; VP16, etoposide.

The diagnosis of primary endometrial YST was a challenge due to its rarity. Microscopically, a reticular pattern coexisted with papillary growth in most cases [9]. The reticular structure comprised a labyrinth of channels lined by primitive cells expanding to form microcapsules with flattened, clear, atypical epithelial cells. Papillary growth showed papillary fibrovascular structures in which a central blood vessel with tumor cells projects into the surrounding space (endodermal sinuses and Schiller–Duval [S‐D] bodies). Occasionally, there is confusion in differentiating a microcystic or endodermal sinus–like structure from a clear cell uterine carcinoma and a papillary structure from uterine serous papillary carcinoma. The pathological pattern of clear cell carcinoma also shows adenoid structures and clear cells; therefore, it is difficult to distinguish it from YST. But clear cell carcinoma usually demonstrates typical hobnail cells without microcapsule structures and S‐D bodies. In addition to morphologic differences, immunohistochemical staining is helpful. AFP is often focally positive in YST, whereas GPC‐3 is more diffusely positive but is a less specific marker [10]. SALL4 is positive in various malignant GCTs, which can be used to distinguish YST from non‐GCTs such as clear cell carcinoma [11]. Although Paired Box Gene 8 (PAX8) and HNF1*β* can be patchy positive in YST, both are typically diffusively positive in clear cell uterine carcinoma [12]. Moreover, Caudal Type Homeobox 2 (CDX2)/Hepatocyte Paraffin 1 (Hep‐par‐1) are strongly positive, and CK20 is focally positive in YST [2]. Staining of the cytoplasm is typically variable for epithelial membrane antigen (EMA) [1]. In our case, negativity for NapsinA further argued against clear cell carcinoma. Ultimately, based on the clinical features, characteristic morphology, and immunohistochemical profile (SALL4+++, AFP++, focal GPC‐3+, focal HNF1*β*+, CK+++, and NapsinA−), our case was diagnosed as a primary endometrial YST.

Given the rarity of primary endometrial YSTs, there is no consensus on the proper treatment of this extremely rare tumor. Surgery combined with adjuvant chemotherapy is currently the primary reported treatment. However, the specific resection range remains controversial, and whether to preserve ovaries or to perform omentectomy still needs study [13]. Standard recommended chemotherapy for ovarian GCTs is bleomycin, etoposide, and cisplatin (BEP) following the 1994 Gynecologic Oncology Group study (GOG protocol 78) [14]. This study demonstrated that three courses of adjuvant BEP regimens after completely resected ovarian GCTs nearly always prevented recurrence, while some patients with recurrent disease can also be treated with high‐dose chemotherapy and stem cell transplantation as a salvage therapy [15]. Among the cases reported on in the literature, all of them underwent surgery, and most patients underwent total abdominal hysterectomy and bilateral salpingo‐oophorectomy treatment, while two [16, 13] retained both ovaries and two [11, 17] retained the right ovary. Twenty‐nine of them were administered chemotherapy, except for two for whom no information was available on chemotherapy. Rossi et al. [16] reported a 30‐year‐old patient (Stage II) who underwent a total hysterectomy, retained bilateral appendages, did not undergo pelvic lymph node dissection, and underwent three BEP regimens after surgery. No recurrence was found after 6 years of follow‐up; Wang et al. [17] reported a 29‐year‐old patient (Stage II) who retained the right ovary and underwent BEP chemotherapy four times after the operation. The follow‐up was 39 months and showed no abnormalities. There is no lesion in the right appendage, and the AFP level is normal. Therefore, whether young patients can retain their ovaries to improve their quality of life is a question worth considering [18]. Previous literature reported that a total of six patients had received adjuvant radiotherapy after surgery, but the prognosis of the six patients was significantly different (5–28 months). Zhang et al. [3] reported a 49‐year‐old patient who underwent total abdominal hysterectomy, bilateral salpingo‐oophorectomy, and internal iliac lymph node dissection. The patient refused chemotherapy and used external radiotherapy. No tumor recurrence was observed after 28 months of follow‐up. Therefore, whether postoperative adjuvant radiotherapy can improve the prognosis of patients remains to be further studied.

Although it remains unclear whether the prognosis of endometrial YST is similar to that of ovarian YST, the above studies could serve as references.

## 4. Conclusion

Primary endometrial YST is an extremely rare and highly aggressive malignancy. Diagnosis relies on characteristic histology (reticular patterns/S‐D bodies) and immunohistochemical markers (SALL4/AFP positivity), which are essential for differentiation from somatic carcinomas like clear cell adenocarcinoma. Our advanced‐stage case (FIGO Stage IVb) underscores its poor prognosis when metastatic. Complete surgical resection combined with platinum‐based chemotherapy (e.g., BEP regimen) remains the cornerstone of management, mirroring ovarian GCT protocols. Serum AFP serves as a critical diagnostic and monitoring tool. Given its rarity, treatment strategies require individualization, and accumulating more cases is vital to establishing standardized guidelines. Early recognition and aggressive multimodal therapy are imperative for survival.

**Nomenclature**
AFP: alpha‐fetoprotein
BEP: bleomycin, etoposide, and cisplatin
CA125: cancer antigen 125
CD30: cluster of differentiation 30
CDX2: Caudal Type Homeobox 2
CEA: carcinoembryonic antigen
CK: cytokeratin
EMA: epithelial membrane antigen
ER: estrogen receptor
FIGO: International Federation of Gynecology and Obstetrics
GCT: germ cell tumor
GOG: Gynecologic Oncology Group
GPC‐3: Glypican‐3
HCG: human chorionic gonadotropin
Hep‐par‐1: Hepatocyte Paraffin 1
HNF1*β*
: Hepatocyte Nuclear Factor 1 beta
IHC: immunohistochemistry
NSE: neuron‐specific enolase
PAX8: Paired Box Gene 8
PET/CT: positron emission tomography/computed tomography
PR: progesterone receptor
SALL4: Sal‐Like Protein 4
S‐D bodies: Schiller–Duval bodies
YST: yolk sac tumor

## Author Contributions

Su‐sheng Shi and Yan Song designed this study and revised the manuscript. Hao Cheng drafted the main text and prepared the figures and table.

## Funding

This study is supported by the National Key Research and Development Program of China (2021YFF1201300).

## Disclosure

All authors read and approved the final manuscript.

## Ethics Statement

Ethics approval and consent was approved by Ethics Committee of Cancer Hospital, Chinese Academy of Medical Sciences, Beijing, China.

## Consent

The patient consent form was signed by the patient.

## Conflicts of Interest

The authors declare no conflicts of interest.

## Data Availability

The data that support the findings of this study are available from the corresponding author upon reasonable request.
